# Cellular and Molecular Mechanisms of CD8^+^ T Cell Differentiation, Dysfunction and Exhaustion

**DOI:** 10.3390/ijms21197357

**Published:** 2020-10-05

**Authors:** Daniel J. Verdon, Matthias Mulazzani, Misty R. Jenkins

**Affiliations:** 1 Immunology Division, The Walter and Eliza Hall Institute of Medical Research, Parkville, VIC 3052, Australia; verdon.d@wehi.edu.au (D.J.V.); mulazzani.m@wehi.edu.au (M.M.); 2 Department of Medical Biology, The University of Melbourne, Parkville, VIC 3052, Australia; 3 Institute of Molecular Science, La Trobe University, Bundoora, VIC 3086, Australia

**Keywords:** T cell exhaustion, chronic viral infections, cancer, immunotherapy, epigenetics, PD-1, inhibitory receptors

## Abstract

T cells follow a triphasic distinct pathway of activation, proliferation and differentiation before becoming functionally and phenotypically “exhausted” in settings of chronic infection, autoimmunity and in cancer. Exhausted T cells progressively lose canonical effector functions, exhibit altered transcriptional networks and epigenetic signatures and gain constitutive expression of a broad coinhibitory receptor suite. This review outlines recent advances in our understanding of exhausted T cell biology and examines cellular and molecular mechanisms by which a state of dysfunction or exhaustion is established, and mechanisms by which exhausted T cells may still contribute to pathogen or tumour control. Further, this review describes our understanding of exhausted T cell heterogeneity and outlines the mechanisms by which checkpoint blockade differentially engages exhausted T cell subsets to overcome exhaustion and recover T cell function.

## 1. T Cell Activation and “Effector” Differentiation—An Overview

T cells are important effector immune cells responsible for inducing cell death of virally transformed or malignant cells, after initiating a highly orchestrated program of differentiation. On initial encounter with mature antigen-presenting cells (APC) bearing their cognate peptide epitope in secondary lymphoid organs, naïve CD8^+^ T cells integrate and accumulate a signal through their TCR/CD3 complex and associated adaptors, and through constitutively expressed co-stimulatory molecules of the CD28, SLAM and TNFRSF (expertly reviewed elsewhere) [1]. This initial “priming” stimulation is augmented by paracrine cytokine signalling from APC (for example interleukin (IL)-12 [2,3], or Type I interferons (IFN)) [4] and paracrine and autocrine IL-2 signalling [5] and allows T cells to initiate a program of rapid and extensive division and differentiation [6]. T cells receiving peptide:MHC-mediated stimulation through the TCR/CD3 complex alone, and in the absence of additional co-stimulation and IL-2, do not progress through repeated cell cycles and fail to generate effector progeny or participate in an immune response. This failure of activation is described as anergy and is a complex process mediated in part by the CDK2 inhibitor and AP-1 antagonist p27kip1, as well as by PTEN, a negative regulator of PI3K [7]. Anergy is not discussed in this review, but is expertly reviewed elsewhere [8].

Activated T cells undergo epigenetic remodelling to enable *de novo* transcription of previously silenced genes for effector functions [9]. The contents of cytotoxic granules, including the pore-forming protein perforin, and an array of serine proteases called granzymes, as well as effector cytokines such as TNFα [10] and IFNγ [11] are produced in a division-linked manner [12,13]. These canonical effector cytokines are pleiotropic. TNFα promotes T cell survival and proliferation [14], and can directly induce necrosis of target cells through a TNFR1-JNK signalling cascade that elicits uncontrolled ROS production [15]. IFNγ signalling serves many functions, including inducing IL-12 production by APC, enhancing phagocytosis and enhancing T cell recognition by upregulation of MHC I and II on target cells [16]. Acquisition of effector function is progressive, beginning after 2–3 divisions, but culminating after 6–8 divisions in murine cells [12] and is dependent on transcription factor network changes, epigenetic remodelling and enhanced translational capacity through increased production of ribosomal subunits. Fully realised effector T cells (T_EFF_) have the capacity to migrate from secondary lymphoid organs (SLO) to areas of tissue inflammation, serially engage and kill target cells, reprogram local tissue resident myeloid cells and produce chemotactic mediators that continue to recruit leukocytes to an area of infection or a tumour.

The interaction between sphingosine-1-phosphates (S1Ps) and their receptors play an essential role in T cell trafficking. Post-activation, T cells transiently down-regulate S1PR1 to render them unresponsive to S1P gradients and trap them in the lymph node (LN) during their signal acquisition phase (~1–4 days), as successive APC contacts are often required for full effector differentiation [17,18]. Following T cell differentiation, S1PR1 expression is restored to allow egress to the periphery along S1P gradients [18]. As naïve CD4^+^ and CD8^+^ T cells divide, they alter their chemokine receptor and adhesion molecule expression profiles, to allow repositioning from the paracortical T cell zone to the lymph node periphery through gain of CXCR3 and CXCR4 [19], then to the systemic circulation with the capacity to traffic to and bind inflamed tissue capillary endothelia through expression of CD44, PSGL-1 and CX3CR1 [20,21,22]. Interestingly, the targeting of effector T cell migration can be directed by the source of matured APC they encounter or route of vaccine administration—for instance, programmed homing back to skin or gut via Cutaneous Lymphocyte Antigen (CLA) or α4β7 integrin expression, respectively [23].

The capacity of T cells to form a broad and functional effector compartment and successfully establish immune memory is essential for both acute clearance of a pathogen, and for protection against future exposure. Following clearance of antigen, expanded CD8^+^ effector T cells massively contract mainly via apoptosis, leaving a small memory population capable of antigen-independent maintenance through responsiveness to homeostatic cytokine signals, self-renewal and robust secondary expansion. Under conditions of prolonged antigen exposure, this canonical naïve-effector-memory spectrum can be perturbed, and T cells instead follow a distinct pathway of differentiation and become functionally and phenotypically “exhausted”.

## 2. The Discovery and Functional Characterisation of T Cell Exhaustion

**Figure 1:**
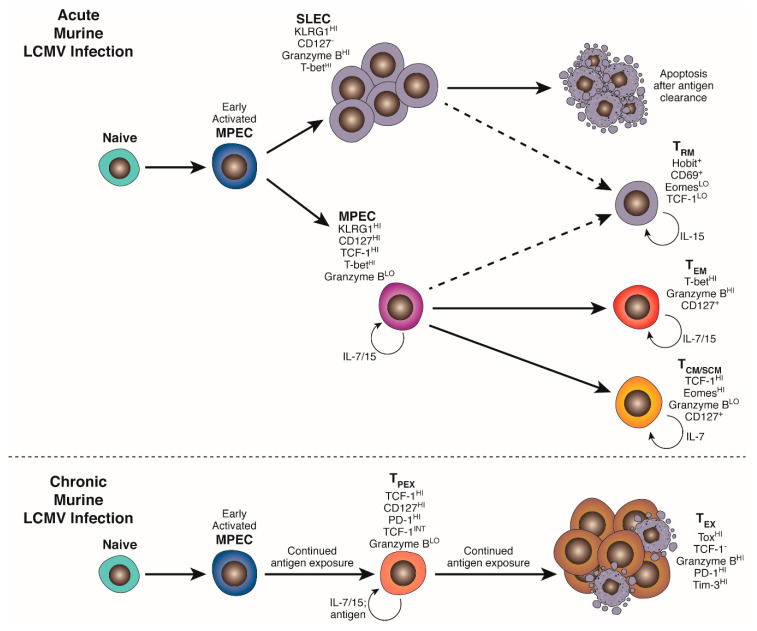
Acute and chronic infection drive distinct programs of CD8^+^ T cell differentiation. Activated naïve CD8^+^ cells initiate a program of metabolic, transcriptional and epigenetic changes that facilitate differentiation into KLRG1^HI^ CD127^neg^ effector and KLRG1^neg^ CD127^HI^ memory precursors (MPEC). In an acute infection, an expanded pool of terminally differentiated cytotoxic effectors (SLEC/T_EFF_) clear infected cells and subsequently contract, leaving behind a heterogeneous pool of MPEC-derived self-renewing stem-like (T_SCM_) and central memory (T_CM_) in secondary lymphoid organs and effector memory (T_EM_) and resident memory (T_RM_) in peripheral tissues to provide protection against secondary exposure to the same pathogen. Chronic TCR stimulation, exacerbated by the absence of appropriate CD4^+^ T cell “help” and co-stimulatory and cytokine signalling, drives an alternative program of differentiation and epigenetic remodeling mediated by NFATc1, BATF, IRF4 and Tox. This gives rise to a TCF-1^+^PD-1^INT^ pool of self-renewing “precursor” exhausted cells carrying a distinct epigenetic signature (T_PEX_) that produce and continually replenish an expanded pool of terminally exhausted progeny (T_EX_) able to partially control, but not fully clear, established infection. T_EX_ maintain the epigenetic signature established in T_PEX_ and exhibit a spectrum of effector function impairment and high expression of a suite of co-inhibitory receptors.

T cell exhaustion was originally described as a functional state induced by chronic antigen exposure and integrating signals from other cell types and the tissue microenvironment. Much of our detailed mechanistic knowledge of effector T cell differentiation and fate has come from comparisons of CD8^+^ T cell phenotype and function in mouse models of Lymphocytic Choriomeningitis virus (LCMV) infection (Figure 1).

Infection with the Armstrong strain of LCMV results in an acute infection that is typically cleared by a broad and effective CD8^+^ T cell response within ~8 days. The Clone 13 strain of LCMV differs from Armstrong in only a few amino acid substitutions in the viral polymerase and glycoprotein, conferring enhanced macrophage tropism and allowing disseminated disease to be established in all visceral organs [24]. As such, Clone 13 LCMV infection is not acutely cleared, and will persist for several months with consistently high viral titres [25]. Despite these differences, key immunogenic epitopes in the viral glycoprotein and nucleoprotein are entirely conserved, allowing direct phenotypic and functional comparison of antigen-matched T cells in each setting [26].

Within the first 4–5 days of acute LCMV Armstrong infection, activated CD8^+^ T cells uniformly acquire effector characteristics, including cytotoxic capacity and IFNγ production, but can be phenotypically partitioned into KLRG1^neg^ CD127^+^ memory precursor effector cells (MPEC) and KLRG1^HI^ CD127^neg^/^LO^ short-lived effector cells (SLEC) [27,28,29]. This distinction is mediated by a complex spectrum of transcription factor interactions, and regulated by strength of TCR-mediated signalling, metabolic profile and inflammatory cytokine exposure (for instance IL-12), discussed in further detail in Section 4 and Section 5. [28,30,31] SLEC briefly retain responsiveness to survival signals from IL-15, but are chiefly reliant on continued antigen exposure for their survival, and have been shown to contract mainly by apoptosis after viral clearance, following kinetics programmed during their initial priming [32,33]. MPEC retain responsiveness to both IL-15 and IL-7 and are not reliant on antigen encounter for survival. These cells persist in SLO after differentiation into long-lived but poorly cytotoxic central-memory (T_CM_) and stem-like memory (T_SCM_) cells capable of robust secondary expansion, and in peripheral tissues and circulation as effector memory cells (T_EM_) retaining both proliferative and cytotoxic capacity, governed by patterns of chemokine receptor and adhesion molecule expression. Although IL-7Rα(CD127) expression and IL-7 responsiveness are crucial for the survival of memory T cells, IL-7 signalling alone is not sufficient for an MPEC—memory transition, as overexpression of CD127 in KLRG1^+^ SLEC does not protect them from contraction [34]. Interestingly, recent cell fate tracking studies suggest that a small proportion of KLRG1^+^ SLEC exposed to intermediate levels of initial stimulation may downregulate KLRG1 while retaining high cytotoxic capacity and contribute to a pool of peripheral tissue resident memory T cells (T_RM_) [35].

Tracking of antigen-specific populations in chronic LCMV infection demonstrated that a classical memory compartment failed to form, revealing instead both progressive deletion of high-affinity CD8^+^ T cells and the persistence of a population of cells that exhibited the partial or complete loss of classical effector functions and contained, but failed to clear virus [26,36,37]. These cells were classified as being exhausted and upon comparison to classical memory or effector cells they exhibit an altered metabolic and transcriptional profile, a unique epigenetic signature, constitutive expression of an array of coinhibitory receptors and an inability to be maintained by homeostatic cytokines [38,39]. The development of this state is dependent on continued high viral antigen load and is exacerbated on CD4^+^ T cell depletion, demonstrating that CD4^+^ T cell help, mediated in part by IL-21 production, plays an important role in LCMV control and effector T cell differentiation [40,41,42]. Seminal early studies tracking peptide-specific recall responses over time demonstrated that CD8^+^ T cell exhaustion is progressive and continual over ~30 days [26,36,41]. Cells sequentially lose the capacity to produce IL-2, TNFα, and finally IFNγ [26,43,44], develop impairment in perforin synthesis [45,46] or effector granule delivery to the immunological synapse [47], lose proliferative potential, and finally exhibit a profound susceptibility to activation-induced cell death [25,48]. Exhausted T cells (T_EX_) gain high constitutive surface expression of co-inhibitory molecules such as 2B4, CD39, CD160, T cell immunoglobulin and mucin domain-containing protein 3 (TIM-3), T cell immunoreceptor with Ig and ITIM domains (TIGIT), and most notably programmed cell death protein 1 (PD-1) through a combination of permissive histone modifications, altered Cp DNA methylation status, enhanced chromatin accessibility and transcription factor expression patterns [39,49,50,51,52,53], further detailed in Section 6 and Section 7. Interestingly, through adoptive transfer experiments both Brooks et al. [54] and Angelosanto et al. [55] defined a window of time whereby T cells exhibiting the early hallmarks of exhaustion could be “rescued” on transfer, 5–8 days after priming, into mice with an ongoing LCMV Armstrong infection, instead developing into a normal effector and memory pool. Conversely, T cells primed during an acute LCMV infection then transferred into Clone 13-infected animals developed an exhausted phenotype, demonstrating that continued antigenic encounter, rather than initial signal integration during priming, was responsible for exhaustion and that an exhausted phenotype was not “fixed”, through epigenetic remodelling, until ~14–30 days after infection. Further, this study and others established that only cells within the KLRG1^neg^ MPEC pool were capable of persisting on transfer and progressing to exhaustion [55,56,57], demonstrating that exhausted cells were not simply a linear phenotypic progression from terminally differentiated effector status and that T_EX_ and T_EFF_ were distinct differentiation lineages. This window of phenotypic flexibility was recently confirmed in a murine model of SV40-T-antigen (TAg)-driven hepatocellular carcinoma, whereby TAg-specific T cells isolated and transferred into naïve hosts at day 8 after initial adoptive transfer and tumour exposure could mount a normal secondary response to challenge by *Listeria monocytogenes* expressing recombinant TAg, while those transferred after 30 days of tumour exposure could not [58].

In addition to other viral infections such as chronic human HIV [59,60], HBV [61,62], HCV [63] and malaria [64], CD8^+^ T cells exhibiting a similar exhausted phenotype have also been described both in murine tumour models, and in tumour-infiltrating lymphocytes (TIL) isolated from solid human tumours [65,66,67,68]. The extent of T cell exhaustion in each setting is antigen-dependent, and correlated with viraemia in HIV and HBV. Interestingly, T_EX_ have been described in several autoimmune conditions, including diabetes mellitus type 1, systemic lupus erythematosus and idiopathic pulmonary fibrosis, and the extent of exhaustion and functional impairment of these cells is inversely correlated with autoimmune damage [69]. Indeed, emerging evidence suggests that CD8^+^ T cell exhaustion appears to be an evolutionary mechanism to facilitate long-term containment of infection without immune-mediated pathology, and despite displaying a limited range of effector functions, exhausted T cells are not senescent. Importantly, although T_EX_ exhibit impairment of canonical effector function, this is variable across models, and T_EX_ heterogeneously retain some capacity to degranulate and kill target cells while also retaining the capacity to patrol infected tissues and produce high levels of chemoattractant chemokines including MIP1α, MIP1β and RANTES [43,70]. Indeed, deletion of CD8^+^ T cells in SIV-infected macaques immediately facilitates a rise in viral titre, suggesting that CD8^+^ T cells unable to completely clear infected cells nonetheless exert sufficient immune pressure to keep chronic viral infection in check and minimize pathology [71,72].

## 3. Heterogeneity and Lineage Relationships in Exhausted T Cells

**Figure 2:**
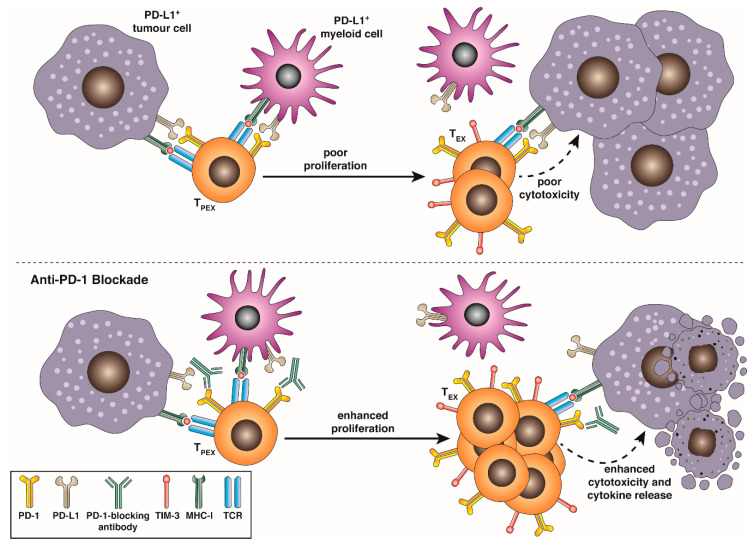
T_PEX_ respond to anti-PD-1/PD-L1 blockade. TCF-1^HI^ intratumoral or virus-specific precursor-exhausted T cells (T_PEX_) expressing intermediate surface levels of PD-1 continually replenish and maintain a pool of cytotoxic granzyme B^HI^ TCF-1^NEG^ PD-1^HI^ TIM-3^HI^ exhausted progeny (T_EX_) when they are stimulated. PD-1:PD-L1 interactions impair activation and proliferation of T_PEX_ and activation cytokine production and direct cytotoxicity of T_EX._ Blockade of these interactions using monoclonal antibodies allows the expansion of a larger tumour- or virus-reactive T_EX_ pool, and enhances direct cytotoxicity against transformed or virus-infected cells.

A major advance in our understanding of T cell exhaustion was the recent discovery that rather than being a homogenous, poorly proliferative compartment incapable of forming immunological memory, exhausted cells could be demarcated into distinct phenotypic and functional subsets in an analogous way to classical memory and effector cells. Following the identification of PD-1 as both an essential marker and mediator of T cell exhaustion and the landmark demonstration that blockade of the PD-1:PD-L1 signalling axis could ameliorate functional exhaustion, restoring effector cytokine production and enabling viral control, several studies provided evidence that responsiveness to PD-1 or PD-L1 blockade was heterogenous, with only a subset of T cells undergoing a proliferative burst (Figure 2). Blackburn et al. [73] demarcated T_EX_ into PD-1^INT^ CD44^HI^ and PD-1^HI^ CD44^INT^ subset, with the PD-1^INT^ CD44^HI^ subset being enriched for cells responsive to anti-PD-1 in clone 13 infection [73,74] and only the PD-1^HI^ CD44^INT^ subset displaying co-expression of PD-1, CD244, CD160 and LAG-3 [74]. Paley et al. found that anti-PD-1 responsive T_EX_ could be identified by high levels of expression of the T-box transcription factor T-bet and by intermediate surface intensity of PD-1. These cells were relatively rare and typically localised to the spleen. By contrast, in this study a larger, poorly proliferative and systemically distributed T_EX_ population expressed intermediate T-bet but high levels of the related T-box transcription factor Eomes, and exhibited high surface co-expression of PD-1, 2B4 and TIM-3 [75]. Utzschneider et al. [76] subsequently demonstrated that the PD-1^+^ population could be divided by expression of TCF-1 (*TCF7*), typically found in naïve, stem-like and central memory cells, but lost in SLEC [77,78]. TCF-1 complexes with **β**-catenin to signal through the Wnt pathway and is a marker of self-renewal capacity [77]. This finding suggested a potential precursor-progeny relationship between rare T-bet^+^/PD-1^INT^/TCF-1^+^ and PD-1^HI^/TCF-1^neg^ subsets. Importantly, subsequent single-cell sequencing studies in in LCMV and murine tumour models have suggested that T-bet and Eomes expression are heterogenous across TCF-1^+^ and TCF-1^neg^ subsets [79,80,81], suggesting that TCF-1 expression is a more accurate means of discriminating precursor and terminally differentiated exhausted cells. Interestingly, although depletion of either subset inhibited control of LCMV viraemia, only ablation of the TCF-1^+^ compartment abrogated responsiveness to PD-1 blockade, suggesting that the proliferative burst provided by disinhibited T-bet^+^ PD-1^INT^ TCF-1^+^ cells was responsible for therapeutic efficacy [76,80,82]. Adoptive transfer studies of both subsets have revealed that only the T-bet^+^ PD-1^INT^ TCF-1^+^ population could survive on transfer into naïve animals and provide protection against subsequent infection or indeed tumour challenge [76,78,80,81]. Further transcriptomic and proteomic analyses have shown that these less differentiated cells exhibit low or no expression of additional co-inhibitory markers such as TIM-3 and 2B4 or cytotoxic mediators such as granzyme B, and express memory associated markers SLAMF6, BCL-6, FOXO-1 and CXCR5, while their terminally exhausted progeny express lack these markers but express high levels of granzyme B and TIM-3. These less-differentiated exhausted cells have been variously described as “stem-like-“ [78,80], “memory-like-” [83,84], “progenitor-” [85], “follicular-” [82] or “precursor-”-exhausted cells [86]. Interestingly, as this self-renewing subset has limited differentiation capacity, only giving rise to terminally exhausted progeny that retain a fixed epigenetic signature of exhaustion, this review utilises the nomenclature recently coined by Kallies, et al. defining PD-1^INT^ TCF-1^HI^ as “precursor exhausted” cells (T_PEX_) and their PD-1^HI^ TIM-3^HI^ progeny as being terminally exhausted (T_EX_) [86]. Single cell RNA sequencing studies through the course of clone 13 infection have recently added complexity to this lineage schema, suggesting that activated T_PEX_ may initially give rise to “transitory” T_EX_ (T_TEX_) that are KLRG1^NEG^, PD-1^HI^, CD101^NEG^ CX3CR1^HI^ (suggesting an early window where T_EX_ are capable of trafficking in response to fractalkine cues). These T_TEX_ develop linearly into KLRG1^NEG^ PD-1^HI^ CD101^+^ CX3CR1^NEG^ terminal T_EX_ [42,87,88]. CD101 is a coinhibitory receptor that has been shown to perturb TCR/CD3-mediated calcium flux to disrupt T cell proliferation [89]. Anti-PD-1 therapy rapidly and transiently increases the frequency of this T_TEX_ population, suggesting they may be the immediate progeny of a T_PEX_ proliferative burst [42,88]. On transfer of T_PEX_ isolated from B16-OVA melanoma into new tumour-bearing mice, Miller et al. found that TCF-1^+^ SLAMF6^+^ TIM3^-^ T_PEX_ could both self-renew and give rise to SLAMF6^+^ TIM3^+^ and SLAMF6^-^TIM3^+^ progeny over 14 days, adding to the model of a phenotypic spectrum among T_TEX_ [85]. By contrast, transferred TCF-1^NEG^ SLAMF6^NEG^ TIM3^HI^ T_EX_ remained phenotypically fixed. The crucial role of T_PEX_ in providing the proliferative burst and producing a large pool of responsive cells following checkpoint blockade is of crucial importance for our understanding of how checkpoint blockade therapies in human tumour settings may operate. This phenomenon has been confirmed in several murine tumour and viral models and has been shown to be CD28 dependent [90], highlighting the importance of retention of co-stimulatory signalling capacity in T_PEX_. Further, TCF-1^+^ PD-1^INT^ and TCF-1^+^ CXCR5^+^ gene signatures in both TIL and circulating T cells have been shown to have predictive prognostic value in human melanoma and lung cancer patients, and in chronic viral disease, highlighting the therapeutic potential of this lineage [81,85,91,92,93,94]. Differential polarisation into T_PEX_/T_EX_ among particular epitope-responsive T cell populations, or TCR-disparate but epitope-matched clones, may be responsible for the clonal replacement observed within TIL following anti-PD-1 treatment, and indeed, Miller et al. found that in B16-OVA TIL, the T_PEX_ compartment contained a far broader spread of TCR clonotypes than did the co-infiltrating T_EX_ compartment [85]. These studies highlight the need to consider each T_EX_ subset when designing therapeutic approaches to target exhausted T cells, and more work is required to understand their dynamic regulation.

## 4. Activation and TCR-Proximal Signal Transduction in Exhaustion

The establishment and progression of T cell exhaustion is dependent on repeated TCR stimulation [57], during which Nuclear factor of activated T cells (NFAT)c1 and NFATc2 are activated by the calcium-dependent phosphatase calcineurin facilitating translocation to the nucleus [95,96]. NFATc1 induces the expression of basic leucine zipper transcription factor (BATF) and interferon regulatory factor 4 (IRF4), and these transcription factors establish a positive feedback cycle that maintains both their and NFATc1 expression [50,97,98]. BATF competitively regulates the expression and functional pairing of AP-1 family members c-fos and c-jun, which normally interact with NFATc1 during SLEC differentiation to tune its control of transcriptional profiles [96]. Further, BATF and IRF4 can directly compete with heterodimeric AP-1 complexes for binding sites, including at the *IL2* locus [99]. c-fos is normally induced by costimulatory signalling and by IL-2, and as such, in situations of repeated TCR ligation where costimulation might be absent and IL-2 production poor, BATF skews the NFAT:AP-1 ratio, and partnerless NFATc1 is able to directly induce the expression of several co-inhibitory receptors, including LAG-3, TIM-3 and PD-1 [95,100]. The *Pdcd1*(PD-1) locus contains a direct NFATc1 binding site −3.7 kb upstream from the promoter. Overexpression of a mutant NFATc1 unable to partner with AP-1 factors exacerbates T cell exhaustion [95], and miR-155 promotes partnerless NFAT activity by degrading mRNA of the AP-1 family member fosl2 [101]. Interestingly, this signalling pathway has been targeted as a means of preventing exhaustion in chimeric antigen receptor (CAR) T cells, a potent adoptive immunotherapy. Lynn et al. [102] demonstrated that CAR constructs exhibiting continuous tonic signalling promoted exhaustion in CAR-T, resulting in loss of IL-2 production and poor cytotoxicity and proliferation on stimulation. Engineering of constitutive c-jun expression alleviated this exhaustion and restored full effector function. Interestingly, even in non-tonically signalling CAR-T cells c-jun expression maintained superior proliferation and anti-tumour efficacy, by countering the regulatory activity of BATF and IRF4 and maintaining an AP-1 mediated transcriptional profile. Utzschneider et al. have recently shown that T_PEX_ early in chronic LCMV (docile) infection could be identified by high expression of BTB and CNC homolog 2 (BACH2), a basic leucine zipper transcription factor that exerts an oppositional transcriptional program to BATF, and by higher levels of accessible chromatin bearing BACH2 binding sites than T_EX_. In this study, BACH2 knockout depleted the T_PEX_ pool and promoted T_EX_ formation, reinforcing the role of BATF in driving T cell exhaustion [103].

NFATc1 signalling induces the expression of nuclear receptor (NR)4A family members and thymocyte selection-associated high mobility group box protein (Tox) family members, transcriptional regulators which have recently emerged as both a key marker and mediator of exhausted T cell phenotype and function [104,105]. In comparisons of acute vs. chronic LCMV infection, Tox expression clearly demarcates T_MEM_ and T_PEX_ at the single-cell sequencing level [18,106]. Tox promotes the progression of an exhausted phenotype and its level is greatest in cells co-expressing high PD-1 and TIM-3 [50,105,107,108]. Interestingly, Tox expression is critical for differentiation and maintenance of both T_EX_ and T_PEX_, as Tox overexpression promotes T_PEX_ formation in chronic LCMV infection [106]. Conversely, Tox knockout mice do not form an exhausted compartment, and on conditional knockout after the establishment of T_PEX_ and T_EX_ populations these cells are rapidly depleted [50]. Therefore, although the mechanism for this depletion is yet to be clarified, Tox knockdown has been shown to reduce surface expression of several co-inhibitory receptors, including PD-1, TIM-3, TIGIT and CTLA-4 and so it is possible that T_PEX_ and T_EX_ are deleted by activation induced cell death as they lose their capacity to “detune” continual TCR signalling [50,108]. In fact, T_EX_ with high-affinity TCR have been shown to be susceptible to deletion by this mechanism during the course of a normal chronic LCMV infection [25]. Indeed, several studies have demonstrated that T_PEX_ and T_EX_ exhibit loss of positive signal adaptors, including TRAF family members [109], and express higher levels of negative signal regulators such as Diacyl-glycerol-kinase; inhibitory phosphatases such as PTPN2 [110]; and E3 ubiquitin ligases [111]—suggesting that exhausted T cells are impaired in accumulating activating signals, which acts both to diminish their immediate effector capacity and protect them from death on stimulation. Interestingly, conditional knockout of *PTPN2* greatly increases the generation, persistence and cytotoxic capacity of TIM-3^HI^ T_EX_ TIL in a murine MC38 model, facilitating enhance tumour clearance [110].

Although several groups have shown that Tox is induced by NFAT, Tox subsequently promotes the expression of NR4A1-3, and these cooperate to maintain Tox expression in an NFAT-independent feedback loop, possibly explaining why Tox expression is maintained in relatively quiescent T_PEX_ [50,104,107]. NR4A family members themselves further promote IRF4 and Runx1 expression, and both Tox and NR4A family members appear to cooperate in order to enforce an exhaustion-specific program of chromatin accessibility [50]. Importantly, Tox directly mediates the acquisition of an exhaustion specific open enhancer region far distal (-23.8kb) to the *Pdcd1* locus and plays an important role in constitutive PD-1 expression on T_PEX_ and T_EX_ [49]. The Tox-NR4A axis has been identified as a potential engineering target in adoptive cell therapies, as both Tox1/2 double knockout [104] and NR4A1-3 triple knockout CAR-T [112] cells exhibited enhanced effector function, cytokine production and tumour clearance in B16-hCD19 tumours, alongside increased chromatin accessibility in genes bound by AP-1 complexes.

Interestingly, although Tox expression was initially identified as being exhaustion-restricted on the basis of acute vs chronic LCMV sequencing comparisons, Sekine et al. have recently demonstrated that Tox expression can also be found in circulating human effector memory T cells specific for latent EBV and CMV antigens [113]. It should be noted that these T_EM_ do also commonly express a poly-inhibitory suite, especially in older individuals, and that cells of these specificities may face periods of antigen resurgence and repeated stimulation on periodic latent viral reactivation [114]. Importantly, Tox expression was not observed in Influenza-virus-specific T_CM_, and was constitutive in circulating HIV, HBV and HCV-specific T cells [113,115]. Similarly, in acute LCMV low and transient expression of Tox can be detected in some cells, consistent with early NFAT-mediated, but not late NR4A-maintained, expression [50]. Nonetheless, in human melanoma and NSCLC TIL Tox expression is highest in cells that exhibit high PD-1, TIM-3 and CD39 co-expression, and Tox expression itself is a predictor of responsiveness to anti-PD-1 therapy [108]. As NFAT, IRF4 and BATF upregulation and signalling are a natural consequence of TCR-mediated signalling under conditions promoting SLEC and T_EX_ formation [39], the precise balance of signals, or frequency and nature of repeated stimulation events required to direct cells down either pathway of differentiation remain to be completely characterized. These studies highlight that a deep understanding of the molecular pathways regulating T cell exhaustion may lead to the identification of further targets for a combined therapeutic approach in T cell redirection immunotherapy, a field still in its infancy.

## 5. Transcriptional Control of Exhaustion

Effector T cell differentiation, memory formation and T cell exhaustion are mediated by a complex interplay of transcription factor, transcriptional coactivator and corepressor networks often involving synergistic or mutually antagonistic signalling pairs.

The T-box transcription factors T-bet (*Tbx21*) and Eomes (*Eomesodermin*) are both induced following TCR ligation and the timing and relative intensity of their expression plays a well-characterised role in the acquisition of canonical effector functions and in the formation of memory T cells [28,29,116,117]. T-bet expression is induced early and promoted by IL-2- and IL-12- and mTOR/AKT-mediated signalling pathways [117,118,119]. T-bet directly promotes IFNγ, TNFα and CXCR3 expression, and promotes IL-12Rβ2 expression, establishing a positive IL-12- and IFNγ-mediated signalling loops [19,28,119,120,121]. Interestingly, T-bet is associated with a loss of CD127 expression in early SLEC [28,29], but also directly binds to and partially represses the *PDCD1* locus, impairing PD-1 expression in both SLEC and in T_PEX_ [70,122]. Partial and complete knockout studies have demonstrated T-bet to be essential in the differentiation of T_EFF_ [28], and the maintenance of both T_PEX_ and T_EX_ in chronic LCMV infection [70]. T-bet levels are lower in MPEC and T-bet is dispensable for a transition to long-lived memory [27,120]. Interestingly, T-bet expression is indirectly maintained by miR-155, which acts to target SHIP-1 (an inhibitory phosphatase that plays a role in T-bet repression) mRNA for degradation [123]. Accordingly, overexpression of miR-155 in chronic LCMV has been shown to promote the survival and activity of T_EX_ [124]. Interestingly, T-bet can induce the expression of the zinc-finger corepressor Zeb2, which binds to and blockades binding sites for the E-box transcription factors E2A and HEB, including CD127 and IL-2 [125]. As such, T-bet and Zeb2 cooperatively enforce effector differentiation, but the role of Zeb2 in T_PEX_ and T_EX_ is less well characterised, and decoupled Zeb2 expression may contribute to T-bet^+^ T_PEX_ retaining expression of memory associated genes.

Eomes is also induced following T cell activation, but early exposure to IL-12 impairs Eomes induction, transiently establishing a T-bet^HI^/Eomes^LO^ window during T cell priming [28,119]. Eomes expression is subsequently driven by FOXO1, TCF-1, Runx3 and Notch1 [118,126,127]. Eomes cooperates with T-bet and Runx1 in maintaining IFNγ expression, and Eomes, Runx3 and Notch1 are each important in binding to and directly promoting perforin and granzyme B expression, whereas T-bet knockout cells show no defect in perforin production [29,118,126,128]. Continued Eomes or Runx1/3 expression in T_EX_ may play an important role in retention of some cytotoxic capacity in these cells, but Eomes also directly upregulates some co-inhibitory receptors, notably TIM-3 [129]. Eomes plays an indispensable role in the transition of MPEC to stem-like and central memory cells, by promoting IL-2Rβ expression to allow responsiveness to homeostatic IL-15, and by driving CD62L and CXCR4 expression to allow localisation to SLO and bone marrow [116,120,130]. Interestingly, a comparison of the transcriptional networks and interaction modules in acute vs chronic LCMV has shown that T-bet and Eomes signalling and interaction modules are distinct and somewhat dysregulated in T_PEX_ and T_EX_ when compared to classical T_MEM_ and T_EFF_ cells, contributing to decoupling of TCF-1 and Eomes expression in T_EX_ [131]. Interestingly, Miller et al. recently demonstrated that in both LCMV Clone 13 infection and in B16 melanoma TIL, high *Eomes* expression was not observed in TCF-1^-^ T_EX_ but high Runx1/3 expression were, mediated by enhanced chromatin accessibility. Runx1/3 were absent from TCF-1^+^ T_PEX_ in this study, suggesting that Runx3 expression may demarcate TCF-1- PD-1^HI^ cytotoxic T_EX_ more accurately than Eomes [85]. As such, the transcriptional network controlled by Eomes, and its role in demarcating subsets of exhausted cells remains an active and incompletely resolved area of investigation.

FOXO1 is essential for induction and maintenance of TCF-1 expression, and for the long-term survival of both T_MEM_ and T_PEX_ compartments through regulation of the CD127 and BCL-2 [132,133]. Indeed, conditional *FOXO1* deletion during chronic LCMV infection led to a rapid loss of T_PEX_ [83,134]. FOXO1 also acts to inhibit expression of cytotoxic mediators and promotes PD-1 expression through binding to a proximal promoter site -1.1 kb from the *Pdcd1* locus [122,134]. As such, expression of FOXO1 may play an important role in establishing the granzyme B^LOW^, PD-1^INT^ phenotype of T_PEX_, through competition with T-bet.

The transcriptional co-repressor BLIMP-1 plays an important role in maintenance of cytotoxic capacity in SLEC, but is not involved in memory T cell formation. Complete or partial BLIMP-1 knockout drives greater accumulation of T_SCM/CM_ in acute LCMV infection or T_PEX_ in chronic LCMV infection, with impairment in effector generation and migration [135,136]. BLIMP-1 expression in chronic LCMV is highest on T_EX_ co-expressing PD-1, 2B4, LAG-3 and CD160, and lowest or absent in T_PEX_, as TCF-1 and BLIMP-1 are mutually antagonistic [78,92,117,137]. Interestingly, while BLIMP-1 acts to directly upregulate the coinhibitory receptor TIGIT, it actually impairs PD-1 expression in a similar way to T-bet, binding in a proximal upstream location at the *Pdcd1* locus and displacing NFATc1 [122,138]. BLIMP-1 acts to recruit histone modifying machinery, including both histone deacetylases and methyltransferases [139]. Exogenous expression of BLIMP-1 in T cells post activation has been shown to silence PD-1 expression though facilitating trimethylation at H3K27 and H3K9, but these repressive marks are lost in exhausted cells even in the presence of BLIMP-1 expression, suggesting an eventual dysregulation of histone methylation machinery. Similarly, BLIMP-1 silences expression of CD27, CD25 and IL-2 through targeted recruitment of HDAC2, resulting in deacetylation and then H3K9 trimethylation at these loci [139].

Finally, the transcriptional regulators inhibitor of DNA binding (ID)2 and ID3 are expressed in effector and memory T cell populations, respectively [140]. These act by binding to and sequestering E-box transcription factor such as E2A and HEB to prevent them controlling a transcriptional program [141], as such ID2 acts to indirectly inhibit the expression of memory related markers and enable effector differentiation. This pattern of expression is conserved in exhaustion, with T_EX_ exhibiting high ID2 expression, and T_PEX_ exhibiting high ID3 expression [80,82,85], although the complex networks of interactivity mediated by ID2 and ID3 in these subsets still need to be fully elucidated.

Overall, notable overlap exists between the transcriptional programs that underlie the T_MEM_-T_EFF_ and T_PEX_-T_EX_ differentiation axes, although the interconnectedness of transcriptional modules in acute and chronic infections appears to be distinct [131].

## 6. The Epigenetic Landscape of T Cell Exhaustion

Despite superficially expressing similar patterns and programs of transcriptional regulators to effector and memory T cells, exhausted T cells exhibit entirely distinct patterns of epigenetic modification and chromatin accessibility with a state-specific epigenetic landscape. The epigenetic signatures of exhausted T cell subsets are typically determined by analysis of permissive and repressive histone acetylation and trimethylation respectively, typically at H3K27 and H3K9, while higher order chromatin accessibility or sequestration into lamina-associated heterochromatin is determined by ATAC-SEQ [51,142]. Recent whole genome studies have determined that T_PEX_ and T_EX_ exhibit several thousand differentially accessible loci when compared to effector or memory T cells [49,143], although this was determined using bulk sequenced populations, and single-cell T_PEX_/T_EX_ analyses and thus a complete understanding of the heterogeneity of the exhausted epigenetic signature remain an active area of investigation. The epigenetic signature of exhausted T cells in chronic LCMV infection appears to be largely the same as that observed in HCV-specific T_EX_ [143]. Interestingly, exhaustion is characterised by a large increase in chromatin accessibility [50,53,85,143], enabling constitutive expression of a various co-inhibitory receptors, and this increased accessibility may be responsible for the differences observed in transcription factor network interactivity described by Doering et al. [131]. The roles of Tox, NR4A and BLIMP-1 in recruiting histone modifying machinery have been previously discussed, and exhausted cells generally exhibit higher levels of DNA Methyltransferase 1 (DNMT1), Methyltransferase 3B (MT3B) and the polycomb repressive complex 2 (PRC2) subunit EZH2 than T_MEM_ or T_EFF_ [144,145]. Repeated TCR stimulation is essential [145,146] in promoting the continuous and progressive accumulation of histone trimethylation from an early stage in chronic LCMV or on tumour exposure, as exhaustion specific methylation patterns are evident in early T_PEX_ and inherited by their T_EX_ progeny [43,53,57,58,145]. Gonheim et al. demonstrated that inhibition of this progressive methylation by DNMT3A knockout preserved effector function even in LCMV Clone 13 infection, and that inhibition of methylation together with PD-1 blockade synergistically enhanced proliferation of virus-specific cells, mediated in part through prevention of methylation at the *myc* locus that would normally act to prevent T_EX_ from initiating cell cycling [145]. Importantly, although PD-1/PD-L1 blockade can alter transcriptional networks and transiently reinvigorate exhausted T cell effector function, several studies have shown that it induces no epigenetic remodelling and as such does not fundamentally alleviate exhaustion [49,145,146,147]. By contrast, treatment of LCMV-specific T_EX_ with HDAC inhibitors during ex vivo restimulation can restore histone modification to a pre-exhausted state and restore effector function on adoptive transfer into LCMV-infected mice [148]. A such, epigenetic modification may play an important future role in ‘reconditioning’ chronic virus-specific- or tumour-isolated T_EX_ in adoptive cellular therapy protocols.

## 7. Extrinsic Mediators of T Cell Exhaustion

### 7.1. Soluble Factors

Extrinsic cell-mediated and soluble signals also contribute to the pathways controlling T cell exhaustion. Metabolic changes in the local environment can promote T cell exhaustion. Tumour cells that deplete their microenvironment of available glucose have been shown to impose metabolic stress on T_EX_ reliant on glycolysis for their metabolic demands. Further, excess potassium flux from necrotic tumour cells [149], buildup of extracellular adenosine [150] and tumour release of kynurenine as a metabolic byproduct of tryptophan metabolism by indoleamine 2.3-dioxygenase (IDO) [151] all impair T cell stimulation, effector function and survival. Therefore, IDO and the Adenosine A2A receptor (A2AR) are active targets of small molecule inhibition to alleviate these suppressive axes [152,153]. One of the main classes of soluble mediators influencing exhaustion are immunosuppressive cytokines. Tumour cells, tumour-associated macrophages and infiltrating myeloid-derived suppressor cells can inhibit T cell function through release of TGFβ and IL-10 - these cytokines repress T-bet expression and upregulate the pro-apoptotic factor Bim. Blockade of either these cytokines or their receptors can synergistically enhance LCMV control in combination with either anti-PD-L1 blockade or vaccination [154,155]. Whether blocking these immunosuppressive cytokines reverses T cell exhaustion or not is yet to be precisely determined. Type I interferons play an essential role in primary T cell activation, and in viral control; however, there is evidence to suggest that prolonged exposure to type I interferon signalling exacerbates T cell exhaustion [78], and that blocking IFNα/β during LCMV Clone 13 infection reversed T cell exhaustion [156].

### 7.2. Coinhibitory Receptor Contribution to T Cell Exhaustion

Tumour cells, tumour-associated macrophages, infiltrating myeloid-derived suppressor cells and, in the liver, infected hepatocytes [157] have all been shown to express an array of ligands for co-inhibitory receptors borne by exhausted cells. These ligand:receptor interactions are key in dampening TCR- mediated and co-stimulatory signalling and imposing T cell dysfunction in situ, and targeted blockade of these interactions has revolutionised our approach to cancer therapy.

Therapeutic targeting of T cell exhaustion has been a topic of intense research for decades, recently celebrated with the 2018 Nobel prize in Physiology and Medicine to Allison and Honjo. Their approach to understand how negative regulators influence T cell function led to the development of immunotherapy as a *bone fide* treatment for cancer. In both cancer and viral infections, several immune checkpoint molecules have since been identified, some of which are amendable to targeted therapy. As outlined in the previous sections, sustained expression of these inhibitory receptors is one of the cardinal features of T_EX_ cells. Most of these receptors are induced by T cell activation, and their co-expression can exert cumulative inhibitory effects on T cells in cancer and chronic viral infection. Therefore, antagonization of these receptors or their ligands has emerged as a valuable strategy in counteracting T cell exhaustion.

Arguably, the most impressive clinical benefit for immune checkpoint inhibition has been obtained by targeting the PD-1/PD-L1 pathway. PD-1, first described in 1992 [158], is a cell-surface receptor inhibiting T cell effector functions. Interaction of PD-1 ligands (programmed cell death 1 ligand 1 (PDL1) and PDL2) with the extracellular domain of PD-1 leads to recruitment of the tyrosine-protein phosphatases SHP-1 and SHP-2 [159,160]. This leads to inhibition of ZAP70, the PI3K-AKT and RAS-MEK-ERK pathways, perturbing both TCR-mediated and CD28-mediated signalling [90] and thereby inhibiting cytokine production, leading to cell cycle arrest, and to decreased expression of cell survival proteins such as Bcl-XL [161]. Although often described in the context of T cell exhaustion, T cells transiently upregulate PD-1 upon activation, and its expression is therefore not entirely exhaustion-specific. The transcriptional and epigenetic control of PD-1 expression is complex, and is expertly reviewed elsewhere [122]. Other cell types have also been found to express PD-1, including B cells, NK cells, NKT cells, some myeloid cells and cancer cells [161]. Additionally, metabolically active and clonally expanding PD-1^+^CD8^+^ cells can be found in the blood of healthy humans and at sites of chronic inflammation, and they are thought to resemble effector memory T cells rather than exhausted cells [162,163]. Interestingly, although expression of PD-1 is a defining phenotypic marker of all exhausted T cells, PD-1 signalling is dispensable in the differentiation of T_PEX_ and T_EX_ and the formation of an exhausted compartment in chronic LCMV, as this occurs normally in PD-1 knockout mice. In fact, in this system progression to terminal exhaustion is exacerbated, and PD-1^-/-^T_EX_ exhibit greater susceptibility to clearance via apoptosis [164], consistent with a role of PD-1 in maintaining exhausted T cell survival by modulating stimulatory signal integration.

Exhausted T cells can also co-express PD-1 together with Lymphocyte-activation gene 3 (LAG-3, also known as CD223) a membrane protein of the Ig superfamily discovered in 1990 [165]. In effector T cells, LAG-3 cross-links with CD3, leading to inhibition of proliferation and cytokine production [166]. In addition, LAG-3 has been shown to have a role as a metabolic regulator [167] and while the intracellular motif necessary for signalling has been discovered in CD4^+^ T cells [168], the exact downstream signalling pathway remains elusive [169]. Similar to PD-1, its expression is not limited to activated T cells (including Tregs [170]), but can also be found on NK cells [165], B cells [171] and pDCs [172]. Its best-known ligand is MHC Class II, to which it binds with higher affinity than CD4 [173,174], selectively binding to stable complexes of peptide and MHC class II [175]. As LAG-3 also regulates CD8^+^ T cell and NK cell function, both of which do not interact with MHC Class II [169], alternate ligands have long been proposed. Indeed, the C-type lectin receptor LSECtin, expressed in the liver and on many tumors, has recently been identified as another ligand of LAG-3 [176]. Additional studies have added galectin-3 [177] and FGL-1 [178] to the list of putative ligands in the context of immunoregulation. The fact that FGL-1 levels in the blood of cancer patients correlates with poor prognosis and resistance to anti-PD-1/B7-H1 therapy suggests FGL-1 as a major contributor to LAG-3 immunoregulation [178]. A soluble form of LAG-3 has also been described [179], resulting from metalloprotease cleavage of the membrane-bound form [180], potentially representing an additional layer of post-translational regulation. Although membrane-bound LAG-3 can inhibit DC function [181], soluble LAG-3 has been described as an activator of antigen-presenting cells [182,183], and it has been used in vaccinations as an adjuvant [184,185]. The clinical activity of soluble LAG-3 (IMP321) has also been tested in renal cell carcinoma [186] and metastatic melanoma [187] with comparably mild side effects. In chronic LCMV infection, LAG-3 expression is co-expressed with PD-1 on exhausted/dysfunctional virus-specific CD8^+^ T cells [188], and it is closely associated to the severity of infection [74]. Similar findings have been described in human cancers such as ovarian cancer [189] and non-small cell lung cancer [190], where LAG-3 expression correlated with PD-1 expression in exhausted/dysfunctional T cells.

A more recently identified immune checkpoint is TIM-3, first described in 2002 [191]. Similar to LAG-3, it is a transmembrane protein belonging to the Ig superfamily. First identified on Th1 and CD8^+^ cytotoxic T cells, it is now known to be expressed also on Tregs [192], NK cells [193], mast cells [194], DCs and monocytes [195], and it can also be expressed by cancer cells such as acute myeloid leukemia stem cells [196,197]. Its role in immunological tolerance [198] has been proposed to be induced by its soluble ligand galectin-9 [199]. Over time, additional ligands have been identified, including membrane-bound phosphatidyl serine (PtdSer) [200] and the soluble ligand high mobility group protein B1 (HMGB1) [201], both of which are mainly important for innate immune responses. The most recently described ligand of TIM-3 is Ceacam-1. It is co-expressed with TIM-3 on CD8^+^ exhausted/dysfunctional T cells, and they can bind both in *cis* and *trans*, leading to induction of tolerance and inhibiting anti-tumor function [202]. TIM-3 mediated signalling seems to be dependent on the balance of intracellular binding partners: In its “permissive” state, the cytoplasmatic tail of TIM-3 is bound by Bat3, which represses the inhibitory function of TIM-3 [203]. Fyn, a known inducer of T cell anergy [204], can bind to the same region as Bat3, potentially competing with it for TIM-3 binding [169,205]. A soluble isoform of TIM-3 has also been described [206]. Similar to the other immune checkpoints, TIM-3 is expressed by a multitude of cells of both the innate and the adaptive immune system, including NK cells [193], DCs [201] and macrophages [207]. Therefore, any therapeutic blockade of TIM-3 could potentially increase the immune function of T cells via both direct and indirect mechanisms.

TIGIT is an Ig superfamily transmembrane receptor first identified in 2009, has been described by several groups under different names, including TIGIT [208,209], Vstm3 [210] and WUCAM [211]. Initially found to be expressed by T cells and NK cells, it has since shown to be expressed by follicular T cells [211] and a functionally distinct subset of Tregs [212]. In T cells, TIGIT binds three targets: CD112, CD113 and CD155 [208,209]. CD112 and CD113 are bound with lower affinity than CD155, and the functional relevance of CD112 and CD113 for T cell dysfunction is not yet clear. TIGIT binding of CD155 (Poliovirus receptor, PVR) on the surface of DCs induces production of the anti-inflammatory IL-10 in these DCs [208]. This process has also been reported after TIGIT binding to macrophage-bound CD155 [213]. In addition to its inhibitory effects on myeloid cells, TIGIT can also intrinsically suppress T cell function. Its affinity to CD155 is higher than CD226 (DNAM-1), which is a known co-stimulatory molecule in T cells [214], thereby competitively inhibiting the immunostimulatory function of CD226. Furthermore, TIGIT can impede the homodimerization of CD226, additionally restricting CD226 signalling [215]. In NK cells it has the cytoplasmatic tail of TIGIT has been shown to transmit inhibitory signals upon ligand binding, inhibiting the PI3K and MAPK pathway [216] and suppression of IFN-γ via NF-κB [217] however, this has not been demonstrated in T cells to date. In T cells, TIGIT activation leads to attenuation of TCR signalling [218]. Preclinical cancer models show that TIGIT is highly upregulated in tumor infiltrating lymphocytes, where it marks a exhausted/dysfunctional T cell population able to produce IL-10 [219]. Also in persistent viral infection, TIGIT is co-expressed with PD-1, Tim-3 and Lag-3 in exhausted, dysfunctional T cells [131,215]. As TIGIT is expressed in multiple cell types, therapeutic TIGIT-blockade might affect T cell function both directly and indirectly.

Several other co-inhibitory receptors have been identified as being expressed on T_EX_ in recent years. B- and T- cell lymphocyte attenuator (BTLA), discovered in 2003 [220], is another transmembrane member of the Ig-superfamily, expressed after T cell activation. Its ligand has been proposed to be HVEM, [221] interestingly a receptor of the TNFR (and not the Ig) family. Similar to other immune checkpoints, BTLA expression is not exclusive for T cells, but also found on B cells, NKTs, DCs, and splenic macrophages [222]. Soon thereafter, CD160 was found to also inhibit T cell function, binding to the same ligand [223]. Both Ig-superfamily receptors (BTLA and CD160) inhibit T cell functions, and together with the stimulatory ligand LIGHT (binding to HVEM) form part of an intricate, bidirectional signalling network (BTLA, CD160, LIGHT, HVEM) [224].

### 7.3. Conclusions and Future Directions

Advances in recent years have shed light on the functional state of T cell exhaustion, which has revealed that it is not an inert and non-functional state, but rather the T cells display a residual level of impeded function. Given the recent evidence that T cell exhaustion can be rescued, it highlights the clinical relevance for understanding the molecular mechanisms driving it. Certainly in the case of chronic infections, exhausted T cells play an important role in controlling disease and, further, immunopathology. It is tempting to speculate that T cell exhaustion has evolved as a strategy to protect us from pathogenic but latent viruses, and thereby a balancing the host-pathogen interaction. But in the case of cancer this work highlights a potential role for targeting and reinvigorating exhausting T cells within a tumour. Sustained expression of inhibitory receptors is one of the cardinal features of an exhausted T cell, and therefore antagonization of these receptors or their ligands has emerged as a valuable strategy in overcoming exhaustion. In fact, antibody-based blockade of the PD-1/PD-L1 interaction leads to durable clinical responses in several types of cancers [225,226,227,228,229] and reviewed elsewhere [230], and has exhibited a therapeutic benefit in chronic HBV and HCV infection [231,232]. These positive responses led to the rapid FDA approval of six checkpoint inhibitor antibodies to date, including 3 anti-PD-L1 and 3 anti-PD-1 antibodies. Although impressive response rates have led to increased overall survival after checkpoint blockade, the majority of patients in these trials did not achieve durable remissions, and PD-1 alone is not sufficient to overcome T cell exhaustion [164]. Therefore, it has been no surprise that combination therapies targeting several inhibitory receptors simultaneously have shown increased response rates in several cancers. The targeting of T cell exhaustion through cytokines has been shown to be effective [233], as has epigenetic targeting and using small molecules to modulate receptor signalling (reviewed in [234]). So, whilst researchers have begun to investigate the metabolic targets, transcriptional regulators and epigenetic targets of T cell exhaustion, the field is still in its infancy, and future studies should focus on combination therapies to rescue T cell function, without compromising T cell persistence. In summary, more research is urgently required into how to overcome a state of T cell exhaustion, and clinical studies should be focused on combination multi-model targeting of the various signals that induce exhaustion for therapeutic intervention of future immunotherapies.

## Abbreviations

APC	Antigen Presenting Cell
BTLA	B- and T- cell lymphocyte attenuator
BATF	Basic leucine zipper transcription factor
CAR	Chimeric antigen receptor
T_CM_	Central-memory T cells
T_EFF_	Effector
T_EM_	Effector memory T cells
T_EX_	Exhausted T cells
HIV	Human Immunodeficiency virus
HBV	Hepatitis B virus
HCV	Hepatitis C virus
IFN	Interferon
IL	Interleukin
IRF4	Interferon regulatory factor 4
LAG3	Lymphocyte activation gene 3 protein
LN	Lymph node
LCMV	Lymphocytic Choriomeningitis virus
MPEC	Memory precursor effector cells
MHC	Major Histocompatibility Complex
NFAT	Nuclear Factor for activation T cells
T_PEX_	Precursor exhausted T cells
PD-1	Programmed cell death protein 1
T_RM_	Resident memory T cells
SLO	Secondary Lymphoid Organ
SLEC	Short-lived effector cells
S1Ps	Sphingosine-1-phosphates
T_SCM_	Stem-like memory T cells
TCR	T cell receptor
TIM3	T cell immunoglobulin and mucin domain-containing protein 3
TIGIT	T cell immunoreceptor with Ig and ITIM domains
T_TEX_	Transitional exhausted T cells
